# Identifying relevant factors for successful implementation into routine practice: expert interviews to inform a heart failure self-care intervention (ACHIEVE study)

**DOI:** 10.1186/s12913-021-06596-w

**Published:** 2021-06-18

**Authors:** Oliver Rudolf Herber, Isabell Ehringfeld, Paula Steinhoff, Amanda Whittal

**Affiliations:** 1 Institute of General Practice (ifam), Centre for Health and Society (CHS), Medical Faculty of the Heinrich Heine University Düsseldorf, Moorenstr. 5, 40225 Düsseldorf, Germany; 2 School of Nursing Science, Faculty of Health, Witten/Herdecke University, Stockumer Str. 12, 58453 Witten, Germany

**Keywords:** Intervention implementation, Relevant factors, Self-care, Heart failure, Behaviour change, Semi-structured expert interviews, Key stakeholders, COM-B model, Normalisation process theory

## Abstract

**Background:**

Adherence to heart failure (HF) self-care behaviours has been found to be effective for alleviating illness symptoms, increasing quality of life and reducing hospital re-admissions and mortality. However, many patients fail to implement on-going self-care into their daily lives. It is therefore crucial to improve the behaviour of HF patients to increase self-care adherence. The aim of this study is to identify relevant factors to successfully implement a complex, theory-based HF self-care intervention into routine practice.

**Methods:**

We conducted semi-structured interviews to obtain key stakeholders’ opinions on previously developed behaviour change techniques for enhancing HF patients’ self-care behaviours, in order to optimise implementation of these techniques in an intervention. The interview topic guide was developed based on the Normalisation Process Theory (NPT), a tool that takes into account the feasibility of implementation and the acceptability to stakeholders. Interviews were analysed using thematic analysis and supported by MAXQDA 2020, a software for qualitative research.

**Results:**

Interview participants included 18 key stakeholders consisting of three crucial groups: clinical experts (*n* = 7), patients (*n* = 3) and high calibre policy makers/potential funders (*n* = 8). The interviews revealed numerous factors to consider for successful implementation of an intervention into routine practice. The findings are presented according to two major categories: (1) themes *within* the NPT framework and (2) themes *beyond* the NPT framework. Themes within the NPT component ‘Coherence’ include three sub-themes: ‘understandability’, ‘value beyond existing interventions’ and ‘perceived benefits’. The NPT component ‘Cognitive participation’ revealed two sub-themes: ‘time resources’ and ‘financial sustainability’. Finally, the NPT component ‘Collective action’ uncovered three sub-themes: ‘need for training’, ‘compatibility with existing practice’ and ‘influence on roles’. A further two themes were identified beyond the NPT framework, namely: ‘structural challenges’ and (2) ‘role of carers’.

**Conclusions:**

Factors identified previously by NPT were validated, but stakeholders further identified relevant aspects beyond NPT. Based on these findings, we suggest the existing NPT framework could be expanded to include a fifth component: questions considering specific environmental factors (contextual considerations). Sensitising researchers to these issues at an early stage when designing an intervention can facilitate its later success.

**Supplementary Information:**

The online version contains supplementary material available at 10.1186/s12913-021-06596-w.

## Background

Heart failure (HF) is a chronic disease, which often leads to premature deaths [1] and significantly lowers the quality of life [2]. Standard treatment as declared in national and international guidelines on HF [3, 4] includes self-care as a crucial part of maintenance. Examples of self-care behaviours comprise daily weighing, medication adherence, regular physical activity and monitoring of fluid intake and symptoms to prevent exacerbations [4]. Adherence to these behaviours has been found effective for alleviating illness symptoms, increasing quality of life and reducing hospital re-admissions and mortality better than drug therapy alone [5], while non-adherence results in the opposite [6]. Still, many patients fail to implement on-going self-care into their daily lives [7]. Therefore, it is crucial to change the behaviour of patients towards better self-care adherence.

Changing health-related behaviour is a complex goal, as behaviours are determined by many factors. Research has shown that simply informing patients about desirable behaviours is insufficient and that applying common sense models hardly results in the desired outcome [8]. Hence, it is necessary to base behaviour change interventions on evaluated scientific models. A review of interventions promoting HF self-care found that only very few studies used explicit theory-based interventions [9]. Thus, it is not surprising that the evidence on the efficacy of such interventions remains inconsistent [10]. There is increasing recognition of the importance of using strong theory to support intervention design [11]. A well-established model for behaviour change is the Capability-Opportunity-Motivation Behaviour (COM-B) model [12]. To our knowledge, the COM-B model is the only behaviour change model that systematically identifies both target behaviours *and* techniques judged and tested to be most effective for changing those behaviours. It has been used successfully in varying scenarios. For instance, to reduce sitting-time in office workers [13] or to change behaviours towards the prevention of spreading COVID-19 [14]. However, the COM-B model has not been used in enhancing self-care in people with HF prior to this study.

Even if an intervention has been based on theory, it will still fail to have a significant impact if local circumstances and sustainable funding have been overlooked [15]. To overcome this problem, the Normalisation Process Theory (NPT) serves as a sensitising tool that takes into account the feasibility of implementation and the acceptability of stakeholders. NPT aims at understanding whether an intervention makes sense to those who will likely work with and be affected by it. It also investigates if there is commitment and engagement to participate in the intervention and if it fits into existing work practices [16]. Hence, obtaining key stakeholders’ insights regarding these parameters in the trial design phase of an intervention is crucial [17] to avoid research waste [18].

In earlier phases of this study, the content for a HF self-care intervention was developed based on the COM-B model. Barriers to HF self-care were identified, and the COM-B model was used to determine which behaviour change techniques (BCTs) were most appropriate to overcome these barriers and improve HF self-care. BCTs are the active components of a full behaviour change intervention. For example, behavioural practice/rehearsal of symptom recognition is one HF self-care BCT that can be part of a complete intervention. The result was a long list of potential BCTs that could be used to develop a full intervention. This list and the process leading to its development has been published previously [19]. It is intended to provide a basis of BCT options that can be used by health professionals with patients to address numerous HF self-care barriers depending on individual patient’s needs. The feasibility and parameters relevant for implementation in individual local contexts can then be assessed, so that the intervention implementation takes into account relevant factors for a given context, and adaptations are made accordingly.

In the German healthcare system, the burden of HF is high, but management of the condition is fragmented and improvements are needed not only in terms of diagnosis and treatment, but also in terms of management (e.g. education and rehabilitation) [20]. The Federal Joint Committee of Germany established the legal framework for an independent Disease Management Programme for HF in August 2018 [21], however, this is not yet on offer and has been delayed [20]. Addressing the need for improved management of HF in Germany would require consideration of context-specific factors, such as the coexistence of private and statutory health insurance, or the fact that HF specialist roles, particularly for nurses, are not consistently integrated into the health care system, thus, it is unclear who should take on some of this responsibility [20].

The aim of this study is to identify relevant factors to successfully implement a complex, theory-based intervention into routine practice [22]. Key stakeholders’ opinions on previously developed BCTs that seek to enhance the adherence of HF patients’ self-care behaviours [19] will be obtained in the light of NPT parameters to optimise implementation. Stakeholders provide specific insight into intervention implementation in the German context, however, these perspectives are extracted to a more conceptual level to examine factors that may be relevant in other contexts beyond Germany.

## Methods

### Determination of sample size

The sample size for the qualitative semi-structured interviews was determined by the concept of information power [23]. Application of this model assists researchers in rationally determining their sample size based on: (1) the aim of the study, (2) sample specificity, (3) use of established theory, (4) quality of dialogue, and (5) analysis strategy. The characteristics of a study can be mapped onto these five factors, which then suggest whether to include a higher or lower number of participants [23].

Since the aim of the study was rather broad and encompassed a variety of topics, a larger purposive sample was needed. The sample specificity was dense as all participants were experts in their respective field, so sufficient information could be gathered by fewer participants – this factor had a large weight in the sample size determination, as expert interviews were conducted with the awareness that they require a smaller sample size. In terms of the use of established theory, this study applied both the COM-B model for designing BCTs and NPT to consider acceptability of potential users and deliverers (less participants needed). Regarding the quality of dialogue, one of the interviewers was a novice researcher with limited experience in conducting qualitative interviews who rarely challenged the participants’ responses (more participants needed). Finally, the analysis strategy was a thematic cross-case analysis including expertise from different points of views (more participants needed). Following our appraisal, we aimed to involve 18 key stakeholders.

### Selection of key stakeholders

Intervention development should be based on meaningful participation of key stakeholders [24]. Therefore, a participatory planning group was established initially to provide input throughout the research process [22]. To obtain a diverse view, the participatory planning group informed the decision to interview stakeholders from three crucial groups: (1) clinical experts, (2) patients, and (3) policy makers/potential funders. Stakeholders were selected by the participatory planning group according to the following criteria: expertise in their field, diverse perspectives, responsibility and authority to facilitate the implementation, influence and commitment [24]. The patient voice had been well-represented in a previous stage of the study, in which we interviewed 31 HF patients about their views on the intervention [19]. Nonetheless, we felt it was important to include patients in this phase of the study as well, to ensure adequate patient involvement as the end users of the intervention. We contacted individual stakeholders via email or telephone and provided them with information about the study. No reward was given for participation.

### Development of interview topic guide

The interview topic guide was developed based on two theoretical approaches and divided into two parts. The questions included in part one were modified based on existing literature [25]. It covers the following four components of NPT: (1) questions considering meaning and sense making (coherence); (2) commitment and engagement (cognitive participation); (3) the work stakeholders do to make the intervention function (collective action), and (4) stakeholders reflecting on the intervention (reflexive monitoring) [25].

Part two of the interview topic guide provided questions that address the specific content of the intervention, as well as detailed information about eight descriptors of an intervention [26]. However, part two is not focused on in this study. The interview questions were adapted to reflect the characteristics of each stakeholder group. Additional probing questions were developed to gain deeper insight. The interview topic guide was developed in English and subsequently translated into German. Finally, the German version was pilot-tested to ensure clarity in the wording of questions (Additional file 1: Appendix).

### Interviews with key stakeholders (data collection)

Once the stakeholders confirmed participation, a date for the interview was scheduled. Originally, all interviews were to be conducted in person [22]. As a response to the coronavirus outbreak, subsequent interviews had to be conducted via video conferencing or by telephone. Stakeholders who were interviewed in person received a copy of the list containing BCTs and the informed consent form via email ahead of the interview. They signed the informed consent form at the beginning of the interview. To those stakeholders who participated remotely after the outbreak, a letter was sent by post containing an informed consent form and a copy of the BCT document. Participants were instructed to sign their informed consent form before interview participation and to return it using a stamped addressed envelope. All methods were carried out in accordance with relevant guidelines and regulations.

The semi-structured interviews were conducted by two members of the research team (AW: psychologist, PS: sociologist) both in German and English. One was experienced in conducting interviews while the other person shadowed in the beginning to learn. In terms of reflexivity, both researchers were conscious that their own assumptions about the intervention could influence respondents’ answers and thus remained neutral in their questioning. As a point of entry into the topic, the following opening question was addressed to clinical experts: “When you think about your HF patients, what self-care measures can they integrate well in their daily lives?” Adjusted opening questions were used for the other two groups. Subsequently, the conversation was directed to the interview topic guide. The interviews were recorded and then transcribed verbatim by a professional transcription service. Once the transcripts were received, the research team performed a quality check by comparing all transcriptions to the audio files. Only very minor divergences were detected.

### Data analysis

The interviews were analysed using thematic analysis [27]. A software package for qualitative research (MAXQDA 2020) was employed [28]. The coding tree was deductively derived from the interview topic guide and complimented by inductive coding of new themes. First, two members of the research team (PS, IE: psychologist) coded the interviews independently and then discussed them. Subsequently, an experienced qualitative researcher (AW) reviewed the coding. Throughout the process the coding tree was continuously adapted to assure the best possible representation of the data. Finally, the coding tree was agreed upon by the three coders (AW, PS, IE) and presented to the principal investigator (ORH: nurse researcher). During the coding process, three members of the research team (AW, PS, IE) met regularly to monitor data saturation based on whether new themes were being generated [29]. Prior to all 18 interviews being conducted, it became evident that no new codes were being generated, however, we continued with all 18 interviews because 1) they had already been arranged and 2) this allowed some further room for the possibility that new codes might still arise.

To ensure consistent coding across all transcripts, a final review of the entire coding tree was performed. Sub-codes that occurred infrequently were merged into broader codes. Individual codes were analysed to identify more abstract themes related to successfully implementing an intervention into routine practice. For each theme, a short definition was written and the content of the code was summarised by comparing and contrasting opinions of different stakeholders. More general takeaway messages were then extracted. Please note that some quotes used below are translations from German into English.

## Results

Interview participants included 18 key stakeholders consisting of three crucial groups: clinical experts (*n* = 7), patients (*n* = 3) as well as high calibre policy makers and potential funders (*n* = 8). The duration of the interviews ranged between 13 and 65 min (*M* = 38.1 min, *SD* = 14.4 min). Participating stakeholders covered a wide range with respect to age and years of professional experience in the current job position. In the case of patients, the number of years since onset of HF was observed. The first two interviews were conducted face-to-face. After the coronavirus outbreak, two interviews were conducted by telephone and fourteen took place via video conferencing (Cisco WebEx). Characteristics of participating stakeholders are summarised in Table 1.

**Table 1:** Characteristics of key stakeholders

Stakeholder group	N	Sex	Years of experience		Age		Interview duration		Interview conduction		
		♂ (♀)	M (SD)	Range	M (SD)	Range	M (SD)	Range	Face to face	Video call	Phone call
Clinical experts i.e., Heart failure specialist nurse (4), cardiologist (2), physician assistant nurse (1)	7	3 (4)	12 (6.3)	4–20	44.9 (7.8)	30–53	40.6 (14.7)	20–60	2	5	0
Patients	3	3 (0)	14.3 (3.8)	10–17	67.0 (11.5)	55–78	26.7 (3.1)	24–30	0	2	1
Policy makers (5) / potential funders(3)	8	4 (4)	13.5 (7.5)	3–25	57.0 (7.1)	44–68	40.1 (15.7)	13–65	0	7	1
Total	18	10 (8)	13.1 (6.3)	3–25	53.9 (11.2)	30–78	38.1 (14.4)	13–65	2	14	2

The interviews revealed numerous factors to consider for successful implementation of an intervention into practice. The findings will be presented according to two major categories: (1) themes *within* the NPT framework and (2) themes *beyond* the NPT framework. Due to space limitations, only key findings are presented. A visualisation of the themes and sub-themes is displayed in Table 2.

**Table 2:** Overview of themes and sub-themes identified through thematic analysis

Themes within the NPT framework			Themes beyond the NPT framework	
Coherence	Cognitive participation	Collective action	Structural challenges	Role of carers
(1) Understandability (2) Value beyond existing interventions (3) Perceived benefits	(1) Time resources (2) Financial sustainability	(1) Need for training (2) Compatibility with existing practice (3) Influence on roles	(1) Cross-sector collaboration (2) Availability of supportive tools (3) Local infrastructure and healthcare system differences	(1) Importance of involving carers (2) Needs of / burden on carers

### Themes within the NPT framework

First, themes are described regarding three of the four components of the NPT framework, namely (1) coherence, (2) cognitive participation and (3) collective action.

#### Coherence

This NPT component refers to the work of sense-making and understanding that individuals and organisations have to go through in order to promote or inhibit the routine embedding of a practice. The following three sub-themes were found: (1) understandability, (2) value beyond existing interventions and (3) perceived benefits.

*‘Understandability’* refers to aspects relating to whether stakeholders were able to understand the intervention (i.e. the list containing BCTs) regarding content, purpose and objective. Many stakeholders agreed that the intervention was generally understandable. Stakeholders emphasised that the list of potential interventions was comprehensive and covered a variety of different aspects regarding self-care for HF patients. Half of the stakeholders found the objective and purpose of the intervention clear, while a few stakeholders struggled to find a clear purpose. Barriers to the understandability of the intervention included use of specific behaviour change terminology, the complexity of the intervention and a wide variety of different intervention techniques. Some stakeholders were confused by the study design, which aimed to use their input to help shape the final intervention. This meant that the form of the eight descriptors were still undefined at the interview stage (e.g. who should deliver the intervention). Hence, some stakeholders found it difficult to understand the intervention and its purpose as the following two quotes highlight:

*‘What you have in mind is clear to me. I just found what you have described as [self-care] measures, sometimes it wasn't clear to me who should do it and how it is all embedded.’* (#17, Policy maker)

*‘When I had the documents, I also skimmed it. I thought … how should something like this be implemented? But I realized that you have a very comprehensive…. catalog of measures that encompasses many individual interventions, which contains suggestions for a very, very large number of… all possible life situations. I was wondering how this should be implemented.’* (#10, Policy maker)

The overall impression was that stakeholders from all three groups were mostly able to understand the intervention. However, some encountered difficulties, primarily due to the stage of the study or intervention complexity.

*‘Value beyond existing interventions’* refers to if and how stakeholders perceived the intervention to have additional value beyond any existing HF interventions they knew of. This theme revealed that many stakeholders perceived the intervention as having added value compared to other HF interventions in two aspects: (1) including family members and carers as an integral part of self-care, and (2) being more comprehensive. Not all aspects of the intervention were perceived as different from existing interventions. Many policy makers and one clinician perceived the intervention as overlapping with existing interventions. For instance, some behavioural techniques also feature on the envisaged Disease Management Programme (DMP) for HF, while others were compared with health services offered by private companies and paid by health insurances.

Generally, some of the behavioural techniques included in the intervention were familiar to the experts. Still, they communicated a strong interest in this intervention and saw added value in having different behavioural techniques combined in one intervention and moving them from the private to the public health care sector.

*‘Perceived benefits’* applies to all three stakeholder groups slightly differently and covers perceptions about whether or not the intervention was perceived as valuable. For patients the focus was on benefits of the intervention for themselves, clinicians’ focus was on benefits for their patients, and policy makers added a health economic perspective. Stakeholders from all groups acknowledged that the intervention would be beneficial for patients and that they would value these benefits. The stakeholders mentioned that patients would benefit from the diversity of approaches reflected in the intervention, i.e. motivational, physical and psychological aspects. Stakeholders primarily emphasised that the intervention would be easy to use for patients, as it contains concrete behavioural instructions such as how to implement physical activity into daily life. According to a few stakeholders, the support provided by the interventionist might help patients to increase their self-efficacy and motivation to perform self-care behaviours.

*‘Patients value that somebody is there to actually care and that they can contact, you know, that there is somebody who has an interest in supporting them with behaviour change because, in my experience, a lot of people are actually keen to change, but maybe have not had the support that they need […] sometimes it is just simple little tricks, hints, tips they need.’* (#5, Clinician)

On the other hand, some characteristics of the intervention were perceived as less beneficial for patients. In some stakeholders’ views, patients would probably need a lot of support to benefit from the intervention because of its complexity and could possibly be overwhelmed by information overload.

*‘I think it is very complex and many patients, we already give a lot of information anyway… I think they quickly get into a situation of being overwhelmed and we have to, I think, work out what the essential points are for the patients.’* (#4, Clinician)

From the health economic point of view, policy makers confirmed that there is a lack of well-evaluated HF interventions in Germany. One policy maker assumed that the intervention could possibly lead to cost savings since it could contribute to avoiding HF decompensation. Yet, this would need to be confirmed by a thorough cost-effectiveness evaluation of the intervention.

Altogether, the intervention was perceived as beneficial for patients by most stakeholders and potentially economically beneficial. It was also perceived as complex, requiring support for patients to ensure they are not overwhelmed.

#### Cognitive participation

This NPT component refers to the work that individuals and organisations have to go through in order to enrol individuals to engage with the new practice. The following two sub-themes were found: (1) time resources and (2) financial sustainability.

*‘Time resources’* refers to aspects in relation to whether patients and clinicians would be willing and able to invest time and energy to participate in the intervention. Patients communicated an interest in improving their HF-related self-care and their willingness to spend time to learn more about it. From the clinicians’ point of view, the ability to allocate time depended on the type of clinician. One clinician claimed that doctors would probably be unlikely to make the necessary adaptations to integrate an additional self-care intervention into their already time-limited treatment routines. HF nurses, however, expressed their motivation to invest extra time to deliver the intervention.

*‘I actually take the time to talk to the patients I am looking after…You just have to adapt a bit to the patient, whether he understands it right away or maybe needs it explained differently or in more detail and then repeated. So - I think the time is definitely there, you can make it longer, but you can also make it short and concise.’* (#1, Clinician)

In general, there was a willingness to invest time resources when possible*.* The willingness existed for patients and realistically, for clinicians who are in a better position to allocate time to such things (e.g. nurses instead of doctors).

*‘Financial sustainability’* refers to aspects related to the sustainable funding of the intervention outside of clinical trials. Policy makers raised different possibilities for financing the intervention, such as applying for funding through existing local programmes or using existing funding structures and policies. The most frequently mentioned source of funding was the future DMP for HF, which would require certain prerequisites in order to be paid for by statutory health insurance companies.

*'The way for funding is to develop a very concrete training programme using these elements [BCTs]. This training programme has to be scientifically evaluated and then general practitioners [GPs] can offer this training programme as part of the disease management programme and it will be paid for.'* (#8, Policy maker)

In general, practical solutions to sustainably funding the intervention included considering how to work with the existing financial options within the local system.

#### Collective action

This NPT component refers to the work that individuals and organisations have to do to enact the new practice. The following three sub-themes were found: (1) need for training, (2) compatibility with existing practice and (3) influence on roles.

*‘Need for training’* refers to opinions on whether staff would need further training to be able to offer the intervention to patients. Stakeholders often expressed that depending on the intervention content, staff may need extensive training to have the necessary competencies to deliver behaviour change interventions. Specific intervention techniques such as motivational interviewing would need to be taught to staff. Distinctions were made, however, between professional groups. For example, HF nurses were seen as more competent to deliver the intervention based on their professional background, while doctors or general nurses without specialisation in HF may need additional training as the following statement illustrates.

*‘[I] would say that staff absolutely need to be trained in what trains the patient, so at least a proper training concept must be followed. So they must have this pedagogical know-how, they must somehow understand the cardiovascular issue and, of course, they must also be trained in how to use these self-management measures.’* (#14, Clinician)

Overall, adequate training of interventionists was raised as a potential need for intervention success.

*‘Compatibility with existing practice’* refers to aspects such as how the intervention could likely be integrated into clinicians’ work practice as well as into patients’ existing self-care practice. Cardiologists practising in the out-patient setting expressed doubts about being able to implement the intervention into their work practice due to an already existing work overload, resulting in short time slots for patients and a large variety of tasks. They therefore could not see an opportunity to integrate the intervention as a whole into their usual practice. They could imagine, however, offering parts of the intervention if it were well organised, e.g. single behavioural techniques in regular patient visits and educational group sessions. Clinicians working in an in-patient setting stated that within a clinical study, staff would be able to accommodate the intervention in their daily work practice, but this would otherwise be difficult due to lack of time. From the patients’ point of view, integrating the intervention into their daily life would generally be feasible, yet perhaps not all aspects of the intervention.

*‘Yes, with the weighing, that definitely fits into everyday life…but as far as salt intake is concerned, you should be careful. Surely this also fits into everyday life, but I don't think all of [the intervention], just some of it.'* (#11, Patient)

Overall, it was highlighted that the intervention as a whole would likely not be possible to accommodate into existing practices, but selected parts of it could.

*‘Influence on roles’* refers to how the intervention would influence the responsibilities and roles of different clinicians. Stakeholder opinions were more varied around this theme. One clinician held the opinion that the intervention would have little influence on the roles, because the main responsibility should remain with the doctor and physician assistants (“*Medizinische Fachangestellte*”) should take on tasks that have been delegated by the doctor. Other opinions were that the intervention would shift more responsibility towards nurses and make patients value their work more.

*‘I think that the role of nurses will be more valued and they will have a closer relationship with patients. You can actually see this quite clearly from the HF nurse that patients love her and then withdraw a little bit of love from the doctor. But it's no problem.’* (#7, Clinician)

Overall, stakeholders did not have much to say about how the intervention might influence current roles, although it was mentioned that nurses might be most appropriate to deliver the intervention.

### Themes beyond the NPT framework

Apart from themes directly related to the NPT framework, two themes plus sub-themes were identified beyond the NPT framework, namely: (1) structural challenges and (2) role of carers.

#### Structural challenges

This theme refers to aspects that need to be considered regarding the system and environment within which the intervention is positioned. It contains three sub-themes: (1) cross-sector collaboration, (2) availability of supportive tools as well as (3) local infrastructure and health care system differences.

*‘Cross-sector collaboration’* refers to the need for health-care sector collaboration for intervention success. Both policy makers and clinicians mentioned that in Germany, the collaboration between in-patient and out-patient care is limited. For the intervention to be successful, they would favour a more collaborative approach for sustainable behaviour change, so that patients receive the same information and care across different settings, e.g. when discharged from hospital and referred to out-patient care.

*‘I think it's like with many things; the more often you hear something, the easier it is to remember and in the outpatient sector, when you have your elective appointment, I think you are mentally receptive to information in a completely different way.’* (#1, Clinician)

Generally, collaboration across health care sectors to ensure consistent patient support was suggested for intervention success.

*‘Availability of supportive tools’* refers to the usefulness of tools that hold the potential to support self-care monitoring when patients are at home. Stakeholders suggested that medical devices such as a weighing scale or a blood pressure monitor could be financed by the health insurance, while other tools such as ergometers or smart tracking devices might need to be financed by the patients themselves, which some patients might not be willing or able to do. Thus, the availability and financing of any supportive tool that is needed for the intervention should be considered to ensure high patient uptake.

*‘Local infrastructure and healthcare system differences’* refers to the issue of having specific services available in some regions and not in others, as well as specific characteristics of a local healthcare system that may affect the success of intervention implementation. Stakeholders highlighted that when planning an intervention to be widely implemented, potential challenges must be taken into account, such as the difference in service availability between rural and urban settings. Additionally, there is inevitable variation in local healthcare systems which is essential to consider for intervention success. For instance, in Germany there is a differentiation between public and private health insurance. Both might be interested in financing a HF intervention for their members, but would have different requirements for adding the intervention to their list of offerings. Another aspect of healthcare system characteristics relates to health professionals. For example, nurses in Germany predominantly undergo traditional hospital-based training as compared to other EU countries where nurses are trained at university level. Besides nurses, physician assistants represent another occupational group in the German health care system that perform not only administrative roles in GP practices, but also have some competencies in patient care. These varying qualifications of healthcare professionals should be considered when deciding who would be best suited to deliver the intervention.

*‘The term nurses in my understanding means nurse with special training who works in Germany in hospitals, and this is a different training than the nurses in Germany who work in clinical practice. The whole training is different. They are trying to bring them closer together in Germany, but the big problem is that, I believe, we have a large deficit in qualifications and in the range of competencies of nurses for the practices compared to the UK.’* (#8, Policy maker)

Overall, local infrastructure and healthcare system characteristics vary greatly and are essential to consider when planning to implement an intervention.

#### Role of carers

This theme refers to the crucial role carers often play for patients with chronic conditions. It contains two sub-themes: (1) importance of involving carers and (2) needs of / burden on carers.

*‘Importance of involving carers’* refers to the fact that partners or other relatives often play a ubiquitous role in a patient’s illness and related behaviours, frequently providing continuous practical and emotional support. Stakeholders therefore considered it essential to involve them in the intervention, as they may assist to implement new behaviours and thus require the information and understanding to be able to do this.

*‘Needs of / burden on carers’* refers to the need to recognise that the role of carers is valuable, but is also a heavy responsibility for the carers themselves. Efforts should therefore be taken to ensure the demand on carers is not overburdening. Including them in the intervention could also provide the support and education they need to fulfil their role without feeling on their own or overwhelmed. Excessive demand on carers was described as follows:

*‘And they are often men who are ill and women who take on this role and then they get so involved in this role and don't even notice it until they are at the end of their tether and say, I can't do it anymore, and the men are always asked how are you doing today and the women are rarely asked because they are not ill, they are the strong partners.’* (#1, Clinician)

Stakeholders generally held the opinion that carers should be included in the intervention, but not overburdened or left alone with responsibilities.

## Discussion

The aim of this study was to identify relevant factors to successfully implement a complex, theory-based intervention into routine practice. To ensure that the intervention can be sustainably implemented, NPT was applied [16, 25]. Thematic analysis revealed several factors within and beyond NPT that experts identified as critical to intervention success.

### Key takeaways from themes within NPT

Stakeholders affirmed that the planned intervention was overall understandable. Although many were familiar with distinct aspects of the intervention, they still showed strong interest in it. This was particularly so because the intervention comprehensively addresses the multi-faceted nature of self-care and includes involving carers (NPT component ‘coherence’). Nonetheless, stakeholders suggested that the complexity of the intervention meant patients would need a lot of support, which could pose a barrier for implementation. The importance of the ‘coherence’ component cannot be overstated for complex interventions. In examining the influence of context on intervention implementation, May et al. [30] emphasise that complexity may lead to intervention failure unless it can be managed effectively.

Both patients and clinicians were willing to invest time resources when possible, but practical limitations must be considered. Some clinicians, such as GPs and cardiologists have a significant lack of time (NPT component ‘cognitive participation’). For intervention implementation to be sustainable, it is therefore important that clinicians who have more time capacity, such as nurses, deliver the intervention. Besides that, stakeholders highlighted that the intervention could be potentially economically beneficial, which could strengthen possible implementation success (NPT component ‘cognitive participation’). Previous literature has corroborated this, highlighting that disease management interventions for HF can lead to a decrease in costs [31]. It was further acknowledged that funding should be integrated into the existing healthcare system to be feasible for routine use, taking into account local characteristics of the system. Germany’s health insurance system, for instance, with the coexistence of private and statutory health insurance, could be a barrier for the implementation of a health intervention that is accessible to the entire population. Other researchers have asserted that to accomplish transferability of an intervention from a primary context (Germany) to another target context (any other country or region), the diverse health care system characteristics must be taken into account [32].

### Key takeaways from themes beyond NPT

All stakeholders favoured a higher collaboration among the health care sectors to ensure consistent intervention support. Research has also shown the value of multidisciplinary interventions in reducing hospital readmissions [31]. Greenhalgh et al. [33] has highlighted that a health intervention is more likely to be adopted when there is ongoing dialogue between different organisations and stakeholders who are part of the process, such as developers, interventionist and end users. Importantly, however, collaboration processes themselves can effect outcomes for patients, health professionals and institutes. Thus, it is essential that such collaboration is carefully organised and of high quality [34].

Stakeholders stressed that structural challenges are essential to consider when planning an intervention. These include, for example, the difference between urban and rural health care infrastructure and specificities of local healthcare systems. These factors are related to the context within which the intervention is implemented. Context has been identified as one of the most important factors for the adoption and the transferability of an intervention [17].

According to Trivedi et al. [35] the involvement of carers contributes substantially to improving self-care and their role should not be underestimated. Likewise, Strachan et al. [36] found that carers – often, but not exclusively members of the family – frequently facilitate three aspects of self-care especially during symptom exacerbation: (1) medication management, (2) sodium reduction including grocery shopping and meal preparation, and (3) symptom recognition. All stakeholders favoured the involvement of carers, who can help to apply the intervention with the caution to avoid them being overburdened [37].

### Strengths and limitations

A strength of this study was the inclusion of key stakeholders in the planning phase to facilitate successful intervention implementation. Nonetheless, some limitations should be noted. First, we were only able to recruit three patients as compared to seven clinicians and eight policy makers/potential funders for involvement as key stakeholders. However, at an earlier stage of the study 31 patients with HF were interviewed to specifically obtain their views on the intervention [19]. Additionally, it was important to focus on clinicians and policy makers/potential funders, since they have more knowledge about the implementation of interventions within health care system infrastructure. Second, both a limit and a strength is the fact that the study sample was exclusively drawn from the German context to account for the idiosyncrasies of the German healthcare system. The strength lies in the identification of relevant factors for HF self-care intervention implementation in Germany. The limit lies in the fact that we aimed with this study to identify factors that may be applicable in other contexts beyond Germany. Thus, although we have extracted generally applicable factors for the implementation of health interventions, it must be acknowledged that the information obtained from key stakeholders was in relation to the German context. In an international context, further challenges might need to be considered to reflect contextual factors. Lastly, although we determined a sample size of participants that should have provided adequate information based on the characteristics, the conclusions of the study do only come from 18 key stakeholders. There was evidence of theme saturation, but there is still a chance that more participants may have brought additional perspectives not captured in this study. Nonetheless, we feel that the study identified valuable factors that can be considered for successful health intervention implementation across countries.

## Conclusion

The findings from this study revealed relevant factors for successful implementation of a behaviour change intervention into routine practice. Factors identified previously by NPT were validated, but stakeholders further identified relevant aspects beyond NPT. These include structural challenges such as local infrastructure and healthcare system characteristics and the significant role carers play for intervention success. Based on these findings, we suggest the existing NPT framework [25] could be expanded to include a fifth component: questions considering specific environmental factors (contextual considerations). The newly added fifth component would consider factors related to the context within which the intervention is implemented. Sensitising implementation researchers to these issues at an early stage when designing an intervention can facilitate its later success.

## Supplementary Information

**Additional file 1: Appendix**: Interview topic guide.

## Data Availability

As the data collection approval for the main study states the data to be available only to the researchers, data and materials collected for this manuscript will not be shared.

## Abbreviations

BCTs: Behaviour Change Techniques
COM-B model: Capability-Opportunity-Motivation Behaviour model
DMP: Disease Management Programme
GP: General practitioner
HF: Heart failure
NPT: Normalisation Process Theory
