# Supplementary material for: Identifying relevant factors for successful implementation into routine practice: expert interviews to inform a heart failure self-care intervention (ACHIEVE study)
Source: BMC Health Serv Res. 2021 Jun 18;21:585. doi: 10.1186/s12913-021-06596-w (PMC8211453; doi:10.1186/s12913-021-06596-w)
Supplement: Supplementary file 1 — Additional file 1: Appendix: Interview topic guide. [file 12913_2021_6596_MOESM1_ESM.docx]

**Appendix 1: Interview topic guide**

**Development of a theory-based intervention to improve self-care adherence in people with heart failure

Guide to semi-structured one-to-one expert interviews**

**Basic key stakeholder information**

**Date** **of Interview:** ___________________ **Location:** ______________________ **Interview duration:** __________

**Interviewer name:** ___________________

**Interviewee name:** ___________________ **Current occupation:** _________________________ **Years of experience*:** ________

**Sex:** ⬜ male ⬜ female ⬜ other **Age:** ______________________

*Years of experience = number of years of professional experience for clinicians or funders/policy makers; years since onset of HF for patients

**PART 1 (NPT): This part of the interview is about your overall impression of the intervention**

Black = Clinicians; Blue = Patients; Purple = Policy makers/potential funders

**Opening question**

Clinical experts: “When you think about your HF patients, what self-care measures can they integrate well in their daily lives?”

Patients: “What self-care measures do you practice in daily life to actively influence your heart failure? Please give us a brief insight.”

Policy makers/potential funders: “What self-care measures do you think HF patients can be sustainably educated on and integrate well in their daily life?”

[*Intervention=long list containing BCTs]

| **NPT component** | **Questions to stakeholders** | **Possible prompts** |
| --- | --- | --- |
| **(1) Coherence** (i.e., meaning and sense making by participants) | - In your opinion, is the intervention* easy to understand? In your opinion, are these suggestions easy to understand? - Is this intervention clearly distinct from other (existing) interventions that you use (e.g. in your organisation)?  Have you ever taken part in something like this?  Do you fund similar, already existing interventions? - Is the objective/purpose of the intervention clear? Is the purpose of these suggestions clear to you?  Is the objective/purpose of the intervention clear? - Do you think that HF patients will benefit from the intervention and value these benefits? What would be the benefits of these suggestions for you personally?  Do you think that this intervention adds value?   (**Optional** – depending on stakeholder –see list) We believe these interventions could lead to cost savings (e.g. through reduced hospital admissions). Would you agree?   - Does the intervention fit with the overall goals and activities of your organisation? Do the suggestions fit with your own goals?   Does the intervention fit with the overall goals and activities of your organisation? | Why or why not?  Why or why not?  Are these suggestions different from that in any way? If yes, is this intervention distinct in any way?  Why or why not?  What is the purpose in your words? Why or why not?  Why or why not?  Do you consider them valuable?  Why or why not? Why or why not?    In what way?  In what way?  In what way? |
| **(2) Cognitive participation** (i.e., commitment and engagement by participants) | - Do you think HF patients will easily understand the intervention? - Would you be prepared to invest time and energy into delivering the intervention? Would you be prepared to invest time and energy into using these suggestions? Would you be prepared to invest (financial) resources to deliver these interventions in the long run? | Why or why not?  Why or why not?  Why or why not?  Why or why not? |
| **(3) Collective action** (i.e., the work participants do to make the intervention function) | - Would you imagine your staff being able to deliver any parts of the intervention?  Would you feel confident using these suggestions on a permanent basis with adequate instruction/training or initial support? **For policy makers like hospital directors*: Would you imagine your staff being able to deliver any parts of the intervention? - How compatible is the intervention with existing work practice within your clinic/hospital? Do you think these suggestions would fit into your daily life? How compatible is the intervention with existing work practice in your organisation? - What influence would the intervention have on division of labour, roles and responsibility between the different clinicians within your setting? What influence would these suggestions have on your spouse/family members/relatives/friends?   What influence would the intervention have on division of labour, roles and responsibility between the staff members within your setting? | What kind of (additional) training would they need to do so?  What kind of (additional) training would they need to do so? |
| **(4) Reflexive monitoring** (i.e., participants reflect on or appraise the intervention) | - How do you, as a clinician, judge the intervention?   How do you, as an expert patient, judge these suggestions?  How do you, as a policy maker/funder, judge the intervention?  If your staff were to deliver any of the intervention would they be willing to provide feedback? If you tried these suggestions would you like to provide feedback on them? If your staff were to deliver any of the intervention would they be willing to provide feedback? | - Why or why not?   Why or why not?  Why or why not? |

Questions are based on: Murray *et al.* (2010). Normalisation process theory: a framework for developing, evaluating and implementing complex interventions. *BMC Medicine* **8**:63

**PART 2 (Descriptors): This part of the interview is about more specific aspects of the intervention**

| **Descriptors** | **Questions to stakeholders** | **Possible prompts** |
| --- | --- | --- |
| **(1) Concrete content and elements of the intervention** (refer to BCT list) | For each BCT: Which BCTs do you think are the most important? For each BCT: What of these suggestions are most important to you? For each BCT: Which BCTs do you think are the most important? | Which ones would you take off the list?   Which ones would you not use?  Which ones would you take off the list? |
| **(2) Characteristics of the person delivering the intervention** (e.g. health professionals, “expert patients”, etc.) | Which person or professional group do you think would be best to deliver the intervention?  From which person or professional would you prefer to receive instructions on these suggestions? Which person or professional group do you think is best to deliver the intervention?  Now consider how their time can be reimbursed to ensure sustainability of the service? | E.g. nurse, doctor, "expert patients", etc.  And why? And why?   E.g. doctor, nurse, "expert patients", etc.  And why? |
| **(3) Characteristics of the target group of the intervention** (e.g. children with HF, adults with HF, NYHA I-IV, etc.) | Which target group do you think this intervention should specifically address? Who do you think the suggestion is most appropriate for? Which target group do you think this intervention should specifically address? | E.g. HF patients with NYHA I-IV; carers; etc.  And why? And why?  E.g. HF patients with NYHA I-IV; carers; etc.  And why? |
| **(4) Location where the intervention should be delivered** (e.g. hospital, clinic, GP practice, community hall, home environment, etc.) | Where do you think the intervention should take place?   Where would you ideally like to receive instruction on something like this? Where do you think the intervention should take place? | E.g. hospital, GP practice, community centre, etc. Any why?  And why?  E.g. hospital, GP practice, community centre, etc.  And why? |
| **(5) The way that the intervention will be delivered** (e.g. group-based, individual approach) | How do you think the intervention should be offered?  How would you prefer to receive something like this?  How do you think the intervention should be offered? | E.g. individual training, group training. And why? E.g. individual training. And why? E.g. individual training, group training. And why? |
| **(6) Format of the intervention** (e.g. lectures, manual, etc.) | In what format do you think the intervention should be offered? In what format would you prefer to receive something like this?  In what format do you think the intervention should be offered? | E.g. classroom instruction, manual, etc.  And why? E.g. classroom instruction.  And why?  E.g. classroom instruction, manual, etc.  And why? |
| **(7) Intensity of the Intervention** (e.g. contact time such as six sessions over three weeks) | What do you think the contact time of the intervention should be? For what amount of contact time would you prefer something like this to be offered? What do you think the contact time of the intervention should be so that time for the interventionist (depending on response from #2) can be reimbursed sustainably? | E.g. contact time 15min, 30min, one hour.  And why? E.g. contact time 15 min.  And why?  E.g. contact time 15min, 30 min, one hour.  And why? |
| **(8) Intervention duration** (e.g. number of sessions over a given period) | How frequently do you think the intervention should be offered? How often would you want something like this to be offered? For how long do you think the intervention should be offered? | E.g. two times per week for one month.  And why? E.g. two times per week  And why? E.g. two times per week for one month.  And why? |

Questions are based on: Davidson *et al.* (2003). Evidence-based behavioural medicine: what it is and how do we achieve it? *Annals of Behavioural Medicine* **26**: 161-171.
